# *MECP2* Duplication Syndrome: AI-Based Diagnosis, Severity Scale Development and Correlation with Clinical and Molecular Variables

**DOI:** 10.3390/diagnostics15010010

**Published:** 2024-12-25

**Authors:** Lourdes Vega-Hanna, Dídac Casas-Alba, Sol Balsells, Mercè Bolasell, Patricia Rubio, Ana García-García, Oscar García-García, Mar O’Callaghan, Ainhoa Pascual-Alonso, Judith Armstrong, MDS Group, Antonio F. Martinez-Monseny

**Affiliations:** 1 Genetics Department, Hospital Sant Joan de Déu, Member of ERN-ITHACA, 08950 Esplugues de Llobregat, Spain; 2 Statistics Department, Fundació de Recerca Sant Joan de Déu, 08950 Esplugues de Llobregat, Spain; 3 Clinical Immunology Unit, Hospital Sant Joan de Déu-Hospital Clínic, 08950 Barcelona, Spain; anapilar.garciag@sjd.es; 4 Servei d’Atenció Ambulatòria, Fundació Aspace Catalunya, 08038 Barcelona, Spain; ogarciag@aspace.cat; 5 Pediatric Neurology Department, Hospital Sant Joan de Déu, 08950 Esplugues de Llobregat, Spain; mariamar.ocallaghan@sjd.es; 6 Fundació de Recerca Sant Joan de Déu, 08950 Barcelona, Spain; 7 Centro de Investigación Biomédica en Red de Enfermedades Raras (CIBERER), Instituto de Salud Carlos III, 28029 Madrid, Spain

**Keywords:** *MECP2*, *MECP2* duplication syndrome, clinical severity scale, facial recognition, artificial intelligence

## Abstract

**Background**: *MECP2* duplication syndrome (MDS) (MIM#300260) is a rare X-linked neurodevelopmental disorder. This study aims to (1) develop a specific clinical severity scale, (2) explore its correlation with clinical and molecular variables, and (3) automate diagnosis using the Face2gene platform. **Methods**: A retrospective study was conducted on genetically confirmed MDS patients who were evaluated at a pediatric hospital between 2012 and 2024. Epidemiological, clinical, and molecular data were collected. A standardized clinical questionnaire was collaboratively developed with input from physicians and parents. Patient photographs were used to train Face2Gene. **Results**: Thirty-five patients (0–24 years, 30 males) were included. Key features in males comprised intellectual disability (100%), hypotonia (93%), autism spectrum disorder (77%) and developmental regression (52%). Recurrent respiratory infections (79%), dysphagia (73%), constipation (73%) and gastroesophageal reflux (57%) were common. Seizures occurred in 53%, with 33% being treatment-refractory. The Face2Gene algorithm was successfully trained to identify MDS. A specific clinical severity scale (MECPDup) was developed and validated, correlating with the MBA (a scale developed for Rett syndrome). The MECPDup score was significantly higher in males (*p* < 0.001) and those with early death (*p* = 0.003). It showed significant positive correlations with age (*p* < 0.001) and duplication size (*p* = 0.044). **Conclusions**: This study expands the understanding of MDS through comprehensive clinical and molecular insights. The integration of AI-based facial recognition technology and the development of the MECPDup severity scale hold promise for enhancing diagnostic accuracy, monitoring disease progression, and evaluating treatment responses in individuals affected by MDS.

## 1. Introduction

The *MECP2* gene encodes methyl CpG binding protein 2 (MECP2), which is involved in various functions, including chromatin architecture, RNA splicing and transcriptional regulation, needed for normal brain development [1,2,3]. Loss-of-function mutations in *MECP2* in females cause classic Rett syndrome (OMIM#312750), while hemizygous mutations in males are typically lethal [4]. However, in some cases, specific MECP2 mutations in males can lead to a spectrum of phenotypes, ranging from severe neonatal encephalopathy to intellectual disability with non-specific features [5,6]. Duplications at Xq28 encompassing the *MECP2* gene result in *MECP2* duplication syndrome (MDS) (OMIM#300260), a rare disorder with fewer than 200 cases reported worldwide, but which is estimated to account for 1% of all cases of unexplained X-linked intellectual disability [7,8].

MDS and Rett syndrome share overlapping features but remain distinct clinical entities. Rett syndrome is characterized by a period normal development between 6 and 18 months, after which there is a regression of neurological abilities, including loss of speech, stereotypic movements, microcephaly, seizures and intellectual disability [9,10]. Males with MDS typically present with a severe neurological phenotype, including early-onset hypotonia, global developmental delay leading to severe intellectual disability, limited or absent speech, autistic features, progressive spasticity, and epilepsy in approximately half of the patients [11,12]. Non-neurological features include gastrointestinal manifestations (e.g., gastroesophageal reflux, constipation, and feeding difficulties), predisposition to infections (especially of the respiratory tract), and genitourinary manifestations (e.g., hypoplastic genitalia, cryptorchidism, or hypospadias), and scoliosis [11,12,13].

Rett syndrome has dedicated assessment tools like the Motor Behavior Assessment (MBA) scale [14], but MDS lacks a specific clinical severity scale that accounts for unique features like motor regression, lower limb hypertonia, and immunological or gastrointestinal involvement. Developing such a tool is crucial for patient monitoring, clinical trials, and understanding genotype-phenotype correlations.

MDS patients exhibit specific facial dysmorphic traits, such as thick hair, midface hypoplasia, a narrow and prominent nasal bridge, thick lower lip, and large prominent ears [12]. While these features could be valuable for diagnosis, they have not been considered sufficient on their own to suggest MDS based on clinical observation [12], but have not been analyzed using pattern recognition tools. Face2Gene is a widely used computer-aided facial analysis tool that employs deep learning algorithms to identify syndromic patterns based on facial images, aiding in the early diagnosis of several genetic syndromes [15,16]. However, its application in MDS has not been comprehensively studied.

The objectives of this study were to develop a specific clinical severity scale tailored to MDS, explore its correlation with clinical and molecular variables, and assess the potential for automated diagnosis using the Face2Gene platform.

## 2. Materials and Methods

### 2.1. Study Design

A descriptive and retrospective study was conducted, including genetically confirmed MDS patients who were evaluated at a tertiary pediatric hospital (Hospital Sant Joan de Déu, Esplugues de Llobregat, Spain) between January 2012 and January 2024. See the overall study framework and data pipeline on Supplemental Figure S1.

### 2.2. Data Collection

A specific checklist was designed to assess the severity, with features annotated according to the Human Phenotype Ontology (HPO) database [17]. The medical records of all patients were analyzed. The patients who were able to travel to our center were clinically evaluated.

### 2.3. Genetic Analyses

DNA was extracted from peripheral blood leukocytes using the Puregene DNA Isolation kit (Gentra System, Minneapolis, MN, USA). Different chromosomal microarray platforms based on comparative genomic hybridization (aCGH) were employed to detect duplications, selected by clinicians at the hospitals where of diagnosis. To confirm the aCGH results and to identify the parental origin of the duplication, multiplex ligation-dependent probe amplification (MLPA) was performed using SALSA MLPA P015-D2, E1, F1, or P245-B1 kits (MRC-Holland, Amsterdam, The Netherlands), all of which target the Xq28 region [18]. The protocol was conducted following the manufacturer’s instructions. Additionally, a custom set of probes was designed to specifically analyze the region that was frequently duplicated in our cohort. The design of these custom probes was based on the “Designing synthetic MLPA probes and probe mixes” manual provided by MRC-Holland. The custom probes targeted specific genes, including Zinc finger protein 275 (ZNF275, OMIM*606957), L1 cell adhesion molecule (L1CAM; OMIM*308840), N(alpha)-acetyltransferase 10 (NAA10; OMIM*300013), Transmembrane protein 187 (TMEM187; OMIM*300059), Interleukin 1 receptor-associated kinase 1 (IRAK1; OMIM*300283), Methyl-CpG-binding protein 2 (MECP2; OMIM*300005), Filamin A (FLNA; OMIM*300017), and Vesicle-associated membrane protein 7 (VAMP7; OMIM*300053). As reference probes, the SALSA MLPA Probemix P300-B1 Reference-2 was used, and the reagents were SALSA MLPA EK from MRC-Holland. The protocol provided by MRC-Holland was followed.

Fluorescence in situ hybridization was performed following the manufacturer’s instructions to determine the chromosomal position of the duplication [18]. For this purpose, MECP2 was tagged with BAC RP11-119A22 (Illumina, San Diego, CA, USA) labelled in the red spectrum, and a MECP2 FISH probe labelled in the red spectrum (Empire Genomics, Williamsville, NY, USA) was used. Additionally, IRAK1 was tagged with an IRAK1 FISH probe labelled in the red spectrum (Empire Genomics). The centromere probe used was Vysis CEP X (DXZ1), which was labelled in the green spectrum (Abbott Laboratories, Chicago, IL, USA).

The X chromosome inactivation (XCI) status of female patients and all carrier mothers was determined by studying the methylation status of the highly polymorphic trinucleotide X-linked androgen receptor (AR) locus, as described by Allen et al. (1992) [18,19]. Inactivation was considered skewed (non-random) if the ratio was >80:20.

### 2.4. Automated Image Analysis

A diagnostic algorithm was developed and trained using facial recognition within the Face2Gene platform (FDNA, Boston, MA, USA; https://face2gene.com, accessed on 9 December 2024). Face2Gene is a widely used tool in the field of syndromic rare diseases, leveraging automated image analysis based on pattern recognition of frontal photographs [15,20].

The 2D image analysis pipeline is composed of several steps. First, the frame of the face is identified using Haar-based cascaded face detection algorithm [21]. Then, 130 fiducial facial points are located using similar local image detectors, each trained individually for a specific point [22,23]. Based on these anatomical points, various local properties are calculated, including distance ratios and local image descriptors of the face. These measurements are used in combination with statistical models known as Bayesian networks [24] to detect dysmorphic features and assess the degree of similarity to the gestalt associated with numerous genetic syndromes for which the system is trained to identify. Additionally, local image information is synthesized to generate a descriptive representation or “gestalt” of the face. This is achieved using vectors of local binary patterns to encapsulate the overall appearance [25]. Finally, a mask is generated to represent the characteristic appearance associated with each syndrome.

For the purpose of this study, the algorithm was developed by training it with frontal facial photographs of both male and female MDS patients, carefully observing any differences. This process was divided into two distinct phases: (I) training of the algorithm within the tool, (II) assessment of distinctness of facial phenotypes.

Phase I:

Initially, the Face2Gene CLINIC application was unable to identify MDS in the patient’s facial photographs as its mathematical algorithm had not yet been trained to recognize this condition. Facial photographs of MDS patients were uploaded to facilitate the training process. Subsequently, a new group of MDS patient images was used to evaluate the performance of the trained algorithm.

Phase II:

To assess the specificity of the facial phenotypes in MDS, a cohort of healthy controls matched in age and gender was selected. Face2Gene RESEARCH allows in silico experiments with user-defined cohorts, and the results are expressed in a confusion matrix for the original sample. Statistical analysis was conducted as described below.

### 2.5. Severity Scale Development and Validation

The specific MDS scale was developed based on the MBA and the main distinctive clinical features of MDS [11,12,13,14]. The MBA provides a more comprehensive evaluation of motor, behavioral, and respiratory dysfunction compared to the Clinical Severity Scale used for Rett syndrome patients [26]. This novel scale was correlated with the MBA and validated for patients with MDS.

Different items were selected by clinical expertise and feedback from parents, developing and modifying 5 items from the MBA scale to make it more representative of MDS. The analysis resulted in a five-factor model, encompassing the following domains: (M) motor dysfunction, including motor regression related with progressive lower limbs hypertonia, ambulation and hand use; (E) presence of epilepsy; (C) functional and social skills, including language and nonverbal communication; (P) pneumological infections; and (D) digestive symptoms. The scale was named “MECPDup scale” after the initials of the main variables and the name of the syndrome itself (Table 1).

Each item was graduated according to the severity of the symptom or the age at which the item was acquired, and if it was lost or never acquired. The scoring system ranges from 0 to 34, with higher scores reflecting a more significant impact on the individual. We establish that MECPDup scores between 0 and 10 indicate mild impairment, scores between 11 and 22 indicate moderate impairment, and scores between 23 and 34 indicate severe impairment.

The scale was validated exclusively in patients who were clinically assessed in person. This process was conducted through the comparison and concordance of recordings of two blinded observers.

### 2.6. Statistical Analysis

Categorical variables are described using counts and percentages, while numerical variables are presented as mean and standard deviation or median and interquartile range, depending on their distribution. The Shapiro-Wilk test was used to assess the normality of numerical variables. Fisher’s exact test was employed to study the relation between categorical variables. Mann-Whitney U test was used to compare nonparametric variables between two groups. Pearson’s correlation coefficient was used to measure the strength of association between two parametric variables, while Spearman’s correlation was utilized for nonparametric variables. We examined the internal consistency (Cronbach’s alpha), and inter-rater reliability of the new scale (Pearson correlation and Interclass correlation coefficient).

For Phase II of the automated image analysis, the area under the curve (AUC) of the receiver operating characteristic (ROC) curve was computed to evaluate the performance of the diagnostic algorithm developed for automated image analysis using the Face2Gene platform. In order to measure the statistical significance, p value random permutation tests and train models were calculated 1000 times.

A two-sided test with a significance level of *p* < 0.05 was considered statistically significant. To perform these analyses, SPSS Statistics for Windows, version 23.0 (IBM Corp., Armonk, NY, USA), was utilized.

### 2.7. Ethical Approval

The study protocol was reviewed and approved by the Research & Ethics Committee of the Hospital Sant Joan de Déu, Barcelona, Spain (internal code: PIC-56-16). The study was conducted in accordance with the Declaration of Helsinki, Good Clinical Practices, and applicable regulatory requirements. All parents and adult patients provided written informed consent, and adolescent patients able to understand the procedure gave their assent prior to patient enrollment.

## 3. Results

### 3.1. Description of the Patient Cohort

A total of 35 patients (30 males) aged between 11 months and 31 years of age (mean age 15.1 ± 6.1 years) were included in the analysis. Among them, 27 had already been described in a previous article [9]. The mean age at diagnosis was 8.1 ± 6.1 years. Among the patients, there were two pairs of brothers (P5–P6 and P10–P11) and a pair of cousins (P21–P22). Four patients died due to respiratory complications: P5 and P6 (brothers) at the ages of 31 and 8 years old, respectively; P2, P3, P9 and P12 died at 11 months old, 12 years old, 6 years old, and 6 years old respectively.

### 3.2. Clinical Features Observed in MDS Patients

The clinical data of the cohort is presented in Table 2 and Supplemental Table S1. The most prominent clinical features in male patients included delayed speech and language development (100%), intellectual disability (100%), and generalized neonatal hypotonia along with delayed ability to walk (both 93%). Stereotypic behavior was observed in 90%, continuing autism in 77%. Recurrent respiratory infections and constipation were present in 79%, together with swallowing difficulties, constipation and gastroesophageal reflux (73%, 73% and 57%, respectively). Seizures were reported in 53%, being treatment-refractory in 33% of the cases, and developmental regression was observed in 52%. Progressive spasticity was found in 27%.

**Figure 1:**
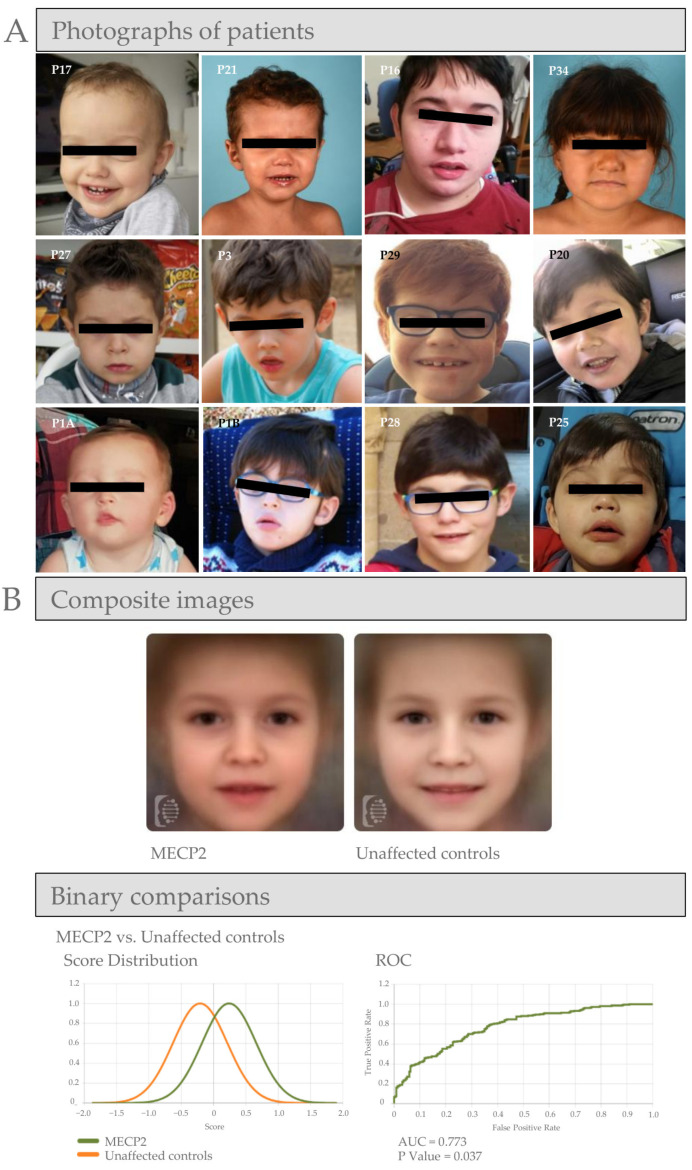
Photographs of patients with *MECP2* duplication syndrome (MDS) and Face2Gene facial recognition analysis of MDS patients. (**A**) Twelve photographs of patients with MDS, illustrating typical facial characteristics: macrocephaly, brachycephaly, dense hair, mild facial hypotonia, hypertelorism, downslanted palpebral fissures, strabismus, midface hypoplasia, prominent ears, nasal tip prominence, narrow nasal bridge, open-mouth appearance, thick lower lip, and dental anomalies. (**B**) Face2Gene facial recognition analysis of MDS patients. Composite images computed from photographs of MDS patients and unaffected controls. Results are displayed as score distribution and receiver operating characteristic (ROC) curves, respectively.

The main dysmorphic features in males are shown in Figure 1. Most of them presented with macrocephaly, brachycephaly, thick and dense hair, hypertelorism, downslanted palpebral fissures, strabismus, facial hypotonia with midface hypoplasia, large ears, prominent tip of nose, narrow nasal bridge, open mouth appearance, thick lower lip and teeth anomalies.

### 3.3. Genetic Analysis

All patients had duplications detected via aCGH, which were confirmed using qPCR and our custom MLPA assay. To determine the origins of the duplications, parents were studied using MLPA, with data confirmed by qPCR and our custom MLPA assay. Among the cases, 11 were de novo, while the remaining 24 were inherited from 22 asymptomatic carrier mothers. We performed FISH to identify the chromosomal positions of duplications. We studied the inactivation status in the 22 carrier mothers and 4 female patients. Among the female patients, 4 had skewed inactivation results: P32 (90:10), P33 (83:17), P34 (84:16), and P35 (100:0).

### 3.4. Computer Facial Recognition Analysis and Proposed Gestalt Picture

The trained algorithm correctly identified 43 facial photographs of patients used for training. Eighteen photographs of new patients with a confirmed diagnosis of MDS were added. In more than 95% of the cases, MDS appeared as one of the top 10 syndrome matches offered by the tool. The other 9 most frequently suggested syndromes by the tool were: Kabuki, Angelman, Kleefstra, KAT6A, Phelan McDermid, Noonan, Pitt-Hopkins, Sotos and Stickler syndromes. The Face2Gene RESEARCH application for facial phenotypic analysis was used to perform binary comparisons and to create ROC curves showing that a recognisable facial pattern in MDS was both distinctive from healthy controls with an AUC of 0.77 (*p* < 0.037) (Figure 1).

### 3.5. Severity Scale Validation

The MECPDup Scale was validated in 27 patients. Internal consistency was assessed through Cronbach’s Alpha test, yielding a value of 0.81. Inter-rater reliability was evaluated using the Pearson Correlation test (0.99) and the Interclass Correlation Coefficient (ICC) (0.938).

We compared the mean scores and standard deviation for both scales. The MBA Scale had a mean score of 11.89 ± 5.73, while the MECPDup Scale had a mean score of 12.63 ± 6.53. A significant correlation was found (*p* < 0.001) with a coefficient of 0.966, indicating a strong linear relationship, though the novel scale consistently produced higher scores.

We also analyzed the impact of 5 modified items (motor regression, language, epilepsy, gastrointestinal symptoms and infections) and the differences between scales using Spearman’s correlation. All correlations were significant (*p* < 0.001) and positive, with higher correlations on MECPDup Scale.

### 3.6. Correlations Between the MECPDup Score and Specific Variables

The correlations between the MECPDup score and specific variables were analyzed across the entire cohort. The MECPDup score was significantly higher in males compared to females (*p* < 0.001) (Table 3). There were no significant differences in age at MECPDup evaluation or duplication size between males and females (*p* = 0.837 and *p* = 0.766, respectively). There was no significant difference between males and females in terms of duplication outside the X chromosome or early death (*p* = 1.000, for both). However, the presence of de novo duplications showed a trend toward significance (*p* = 0.095), with slightly higher occurrences in females.

In males, the MECPDup score showed significant positive correlations with age at MECPDup evaluation (R = 0.720, *p* < 0.001) and duplication size (Rho = 0.370, *p* = 0.044) (Supplemental Figure S2). However, there were no significant differences in the MECPDup score based on de novo duplications or duplication outside the X chromosome (*p* = 0.321 and *p* = 0298, respectively). Additionally, males with early death had significantly higher MECPDup scores compared to the rest of the group (*p* = 0.003).

In females, no significant correlations were observed between the MECPDup score and age at MECPDup evaluation, duplication size, or biased X chromosome inactivation (*p* = 0.511, *p* = 0.741, and *p* = 0.564, respectively) (Table 2). Other analyses were not possible because of the small sample size.

## 4. Discussion

### 4.1. Clinical Characterization of MDS

The present study provides a comprehensive clinical and molecular description of MDS, expanding the understanding of this disorder. Consistent with previous research, our findings confirm that MDS presents with a more severe phenotype in males [27], characterized by developmental delay, intellectual disability, autism spectrum disorder, dysmorphic features, and epilepsy. Notably, developmental regression was observed in 40% of cases, often coinciding with epilepsy onset. Additionally, gastrointestinal manifestations, respiratory infections, and genitourinary issues were identified as common features in MDS patients.

### 4.2. Facial Pattern Recognition Technologies

Emerging facial pattern recognition technologies, such as Face2Gene, have proven valuable across a broad range of genetic conditions [15,16]. In the case of MDS, where dysmorphic features may be subtle, leveraging diagnostic support tools that employ next-generation phenotyping is crucial for accurate diagnosis. Our research has facilitated the training of Face2Gene to discriminate and suggest MDS, even in the absence of multisystem involvement and supplementary Human Phenotype Ontology (HPO) [17]. By enabling early suspicion through advanced pattern recognition, our approach facilitates more timely genetic investigations and shortens the diagnostic journey.

### 4.3. Development of the MECPDup Clinical Severity Scale

The development of a dedicated clinical severity scale for rare diseases like MDS holds significant importance in clinical practice. Clinical severity scales serve as outcome measures in clinical trials, help identify genotype-phenotype correlations, and facilitate objective patient progression monitoring [14]. The MECPDup scale addresses a critical knowledge gap by providing a clinical severity scale specifically designed for MDS. In this study, we compared the MECPDup scale with the MBA scale, which is specific to Rett syndrome, a condition that shares overlapping features with MDS but remains a distinct clinical entity. Rett syndrome patients commonly present with respiratory disturbances, hand stereotypies, progressive microcephaly, and autonomic symptoms at a higher frequency. In contrast, two key features of MDS, recurrent respiratory infections and gastrointestinal symptoms, are not evaluated by the MBA scale [26].

The MECPDup scale would enable a more precise assessment of symptomatology in affected individuals, promoting objective communication, and potentially facilitating the establishment of standardized protocols for universal follow-up among healthcare professionals. Our findings provide compelling evidence that the MECPDup scale is equally sensitive and reliable in assessing MSD patients compared to the MBA, while offering the added benefit of a reduced item count. These results affirm the potential of the MECPDup scale as a valuable tool for comprehensive evaluation and monitoring of individuals with MDS, providing clinicians with a reliable and efficient instrument for clinical decision-making.

The significant positive associations between age at MECPDup evaluation and MECPDup score in males underscore the potential of the scale to serve as a valuable tool for monitoring disease progression and evaluating response to future therapies in individuals affected by MDS. Recognizing its potential for progression, the MECPDup scale offers a dynamic assessment, allowing for tracking changes over time and providing valuable insights into the potential progression of the disease. Recent outcomes from MDS animal models show the rapid progress of gene therapy towards practical implementation [28,29,30], underscoring the need to establish tools and protocols before clinical testing and application.

### 4.4. Genotype-Phenotype Correlations in MDS

Establishing a robust genotype–phenotype correlation in MDS has been challenging due to the lack of large cohorts and long-term studies. Identifying both the genotype-phenotype correlation and the molecular pathological basis is not straightforward, given the presence of a specific duplication in each patient containing an exclusive set of specific genes inserted at different genomic loci. Previous studies hypothesized that duplications located outside of chromosome X may serve as markers of disease severity, and skewed X chromosome inactivation in females might be linked to less severe phenotypes [18].

Our study found a significant correlation between the MECPDup score and duplication size in males, which aligns with previous research by Peters et al. who reported a correlation between gene dosage and severity measured with the CSS used in Rett syndrome [31]. However, for other factors such as localization of the duplication outside the X chromosome in males and skewed X chromosome inactivation in females, we did not observe significant associations with the MECPDup score. These non-associations may be attributed to the limited sample size, which may have hindered the detection of subtle correlations.

### 4.5. Study Limitations

Our study highlights the need for future investigations with larger cohorts to adequately explore genotype-phenotype correlations. Other limitations include the heterogeneity of the sample from different centers, the fact that not all patients were evaluated in person at the same time, and the lack of photographs of all patients at the same age.

## 5. Conclusions

This study offers a comprehensive phenotypic characterization of patients diagnosed with MDS, conducted within a single medical facility. The integration of AI-based facial recognition technology and the development of the MECPDup severity scale hold promise for enhancing diagnostic accuracy, monitoring disease progression, and evaluating treatment responses in individuals affected by MDS. Future research should focus on several key areas to advance the understanding and management of MDS. Larger, multicenter cohorts are needed to further validate the MECPDup severity scale across diverse populations and explore genotype-phenotype correlations. Longitudinal studies are essential to track disease progression over time, allowing the refinement of clinical severity assessments and better understanding of natural history. These advancements are vital for improving patient care and facilitating future therapeutic strategies in the management of this rare neurodevelopmental disorder.

## Figures and Tables

**Table 1:** MECP2Dup scale. The analysis resulted in a five-factor model, encompassing the following domains: (M) motor dysfunction, including motor/independent sitting, ambulation and hand use; (E) epilepsy; (C) communication skills; (P) pneumological infections; and (D) digestive symptoms.

	Manifestation	Score	Definition
(M) Motor	Motor regression/lower limbs hypertonia	0	No hypertonia
		1	Mild hypertonia, slightly affects walking
		2	Moderate hypertonia, needs light walking support
		3	Severe hypertonia, no walking regression
		4	Extreme hypertonia with walking regression
		5	Extreme hypertonia with global neurological regression
	Ambulation	0	Acquired <18 months/Apraxic gait
		1	18 months ≤ walks alone ≤ 30 months
		2	Walks alone > 30 months
		3	Walks alone > 50 months or walks with help
		4	Lost
		5	Never acquired
	Hand use	0	Acquired & conserved
		1	Holding of objects acquired on time (by 6–8 months) & partially conserved
		2	Holding of objects acquired late (>10 months) and partially conserved
		3	Holding of objects acquired & lost all acquisitions
		4	Never acquired
(E) Epilepsy	Epilepsy/Seizures	0	Absent
		1	<monthly
		2	<weekly to monthly seizures
		3	Weekly
		4	Infantile spasms
		5	Treatment refractory
(C) Comunnication skills	Language	0	Preserved, contextual
		1	Short phrases only
		2	Single words
		3	Vocalization, babbling
		4	Lost
	Nonverbal Communication	0	Preserved & propositive (points consistently with finger or eyes)
		1	Good eye contact maintained (≥30 s)
		2	Intermittent eye contact (5 s to <30 s)
		3	Infrequent eye contact (<5 s)
		4	Lost and not regained
		5	None
(P) Pneumology	Infections	0	Absent
		1	Recurrent infections
		2	Recurrent infections that requires hospital admission
		3	Recurrent infections and need for immunoglobulin administration
(D) Digestive	Gastrointestinal symptoms	0	Absent
		1	Unique affectation (constipation, gastrointestinal reflux or dysphagia)
		2	Presence of two symptoms (constipation, gastrointestinal reflux or dysphagia)
		3	Total affectation (constipation, gastrointestinal reflux and dysphagia)

**Table 2:** Summary of clinical and molecular data, including the MECPDup score.

	Males	Females
**Demographic variables**		
Age at MECPDup evaluation (years) *	5.5 (3.0–12.3)	6.0 (3.8–13.5)
**Clinical variables**		
Generalized hypotonia (HP:0008935)	28/30 (93.3%)	0/5 (0.0%)
Delayed ability to walk (HP:0031936)	28/30 (96.3%)	0/5 (0.0%)
Progressive spasticity (HP:0002191)	8/30 (26.7%)	0/5 (0.0%)
Ataxia (HP:0001251)	15/29 (51.7%)	0/5 (0.0%)
Intellectual disability (HP:0001249)	30/30 (100%)	3/5 (60.0%)
Developmental regression (HP:0002376)	15/29 (51.7%)	1/5 (20.0%)
Delayed speech (HP:0000750)	30/30 (100%)	5/5 (100.0%)
Absent speech (HP:0001344)	13/30 (43.3%)	0/5 (0.0%)
Stereotypic behavior (HP:0000733)	27/30 (90.0%)	4/5 (80.0%)
Autism (HP:0000717)	23/30 (76.7%)	3/5 (60.0%)
Anxiety (HP:0000739)	15/30 (50.0%)	3/5 (60.0%)
Compulsive behaviors (HP:0000722)	17/30 (56.7%)	3/5 (60.0%)
Bruxism (HP:0003763)	22/30 (73.3%)	1/5 (20.0%)
High pain tolerance (HP:0010832)	10/30 (66.7%)	1/5 (20.0%)
Sleep disturbances (HP:0002360)	19/30 (63.3%)	1/5 (20.0%)
Recurrent respiratory infections (HP:0002205)	23/29 (79.3%)	2/5 (40.0%)
Seizures (HP:0001250)	16/30 (53.3%)	2/5 (40.0%)
Treatment-refractary seizures (HP:0032867)	10/30 (33.3%)	0/5 (0.0%)
Gastroesophageal reflux (HP:0002020)	17/30 (56.7%)	0/5 (0.0%)
Constipation (HP:0002019)	22/30 (73.3%)	2/5 (40.0%)
Swallowing difficulties (HP:0002015)	22/30 (73.3%)	0/5 (0.0%)
Scoliosis (HP:0002650)	10/30 (33.3%)	0/5 (0.0%)
Cryptorchidism (HP:0000028)	6/29 (20.7%)	0/5 (0.0%)
Hypogenitalism (HP:0003241)	2/29 (6.9%)	0/5 (0.0%)
MECPDup score *	18.0 (12.0–22.0)	5. (3.0–6.5)
**Genetic variables**		
De novo duplication	6/30 (20.0%)	2/5 (40.0%)
Duplication outside the X chromosome	3/29 (10.3%)	0/5 (0.0%)
Duplication size (Mb) *	0.5 (0.4–1.0)	0.6 (0.3–8.0)

* Median, P25 and P75.

**Table 3:** Analysis of gender-specific correlations between the MECPDup score and specific variables.

Variables of Interest	MECPDup Score
Analysis in males	
Age at MECPDup evaluation (years)	R = 0.720 *p* < 0.001 (Pearson’s test)
Duplication size (Mb)	Rho = 0.370 *p* = 0.044 (Separman’s test)
De novo duplication	Yes (N = 24) = 18.0 (13.0–22.8) * No (N = 6) = 13.0 (11.5–21.3) * p = 0.321 (M-W)
Duplication outside the X chromosome	Yes (N = 3) = 17.5 (11.8–22.0) * No (N = 26) = 18.0 ** p = 0.298 (M-W)
Early death	Yes (N = 5) = 24.0 (20.5–29.5) No (N = 25) = 15.0 (11.5–20.5) *p* = 0.003 (M-W)
Analysis in females	
Age at MECPDup evaluation (years)	Rho = 0.395 *p* = 0.511 (Spearman’s test)
Duplication size (Mb)	Rho = 0.205 *p* = 0.741 (Spearman’s test)
Biased X chromosome inactivation (highest proportion)	Rho = −0.564 *p* = 0.322 (Spearman’s test)
De novo duplication	***
Duplication outside the X chromosome	***
Early death	***

M-W, Mann-Whitney U test. * Median, P25 and P75. ** P25 and P75 were not calculated due to the group size of 3. *** *p* was not calculated because the subgroups are too small or empty.

## Data Availability

The original contributions presented in the study are included in the article, further inquiries can be directed to the corresponding authors.

## Supplementary Materials

The following supporting information can be downloaded at: https://www.mdpi.com/article/10.3390/diagnostics15010010/s1, Figure S1: Overall study framework and data pipeline.; Figure S2: Scatter plot showing the correlation between the MECPDup score and age in males; Table S1: Clinical and molecular data of each patient.
