# Supplementary material for: MECP2 Duplication Syndrome: AI-Based Diagnosis, Severity Scale Development and Correlation with Clinical and Molecular Variables
Source: Diagnostics (Basel). 2024 Dec 25;15(1):10. doi: 10.3390/diagnostics15010010 (PMC11720083; doi:10.3390/diagnostics15010010)

Supplemental Table S1. Clinical and molecular data of each patient.

Patient	1	2	3	4	5	6	7	8	9	10	11	12	13	14	15	16	17	18	19	20	21	22	23	24	25	26	27	28	29	30	31	32	33	34	35
Sex	M	M	M	M	M	M	M	M	M	M	M	M	M	M	M	M	M	M	M	M	M	M	M	M	M	M	M	M	M	M	F	F	F	F	F
Age (years)	12	0.9	12	1.3	24	8	11	2.8	6	12	9	4	24	7	20	14	2	2	5	4	3	2	13	3	4	5	5	17	15	3	15	6	12	6	1.6
**Clinical variables**																																			
Generalized hypotonia (HP:0008935)	+	+	+	+	+	+	+	+	+	-	+	+	+	-	+	+	+	+	+	+	+	+	+	+	+	+	+	+	+	+	-	-	-	-	-
Delayed ability to walk (HP:0031936)	+	+	+	+	+	+	+	+	+	+	+	+	+	-	+		+	+	+	+	+	+	+	+	+	+	+	+	+	+	-	-	-	-	-
Progressive spasticity (HP:0002191)	+	-	-	-	-	+	-	+	+	-	-	-	-	-	-	+	-	-	-	-	-	-	-	-	-	-	+	+	+	-	-	-	-	-	-
Ataxia (HP:0001251)	+	-	+	-	+	+	+	-	-	+	+	+	+	-	-		+	na	-	-	-	+	+	-	-	+	-	+	+	-	-	-	-	-	-
Intellectual disability (HP:0001249)	+	+	+	+	+	+	+	+	+	+	+	+	+	+	+	+	+	+	+	+	+	+	+	+	+	+	+	+	+	+	+	+	-	+	-
Developmental regression (HP:0002376)	+	+	-	-	+	+	+	+	+	na	-	+	+	-	+	+	-	+	-	-	-	-	+	-	-	+	-	-	+	-	+	-	-	-	-
Delayed speech (HP:0000750)	+	+	+	+	+	+	+	+	+	+	+	+	+	+	+	+	+	+	+	+	+	+	+	+	+	+	+	+	+	+	+	+	+	+	+
Absent speech (HP:0001344)	+	+	+	+	+	+	-	-	+	+	+	+	+	-	+	-	-	-	-	-	-	-	-	-	-	-	-	-	+	-	-	-	-	-	-
Stereotypic behavior (HP:0000733)	+	+	+	-	+	+	+	+	+	+	+	+	+	-	+	+	+	+	+	+	+	+	-	+	+	+	+	+	+	+	+	+	+	+	-
Autism (HP:0000717)	+	+	+	-	+	+	-	-	+	-	-	+	+	-	+	+	+	+	+	+	+	-	+	+	+	+	+	+	+	+	-	+	+	+	-
Anxiety (HP:0000739)	+	+	+	-	+	+	+	-	+	+	+	+	+	-	-	+	-	-	+	-	-	-	+	-	-	-	-	-	-	+	+	-	+	+	-
Compulsive behaviors (HP:0000722)	+	+	+	-	+	+	+	-	+	+	+	-	+	-	-	+	-	-	+	-	-	-	+	-	+	+	-	-	+	+	+	-	+	+	-
Bruxism (HP:0003763)	+	+	+	-	+	+	+	-	-	+	+	+	+	+	-	+	-	+	+	+	-	-	+	+	+	+	+	-	+	+	-	-	-	+	-
High pain tolerance (HP:0010832)	+	+	+	-	+	+	+	-	+	-	+	+	+	-	-	+	-	+	+	+	-	-	+	-	+	+	-	+	+	+	-	-	+	-	-
Sleep disturbances (HP:0002360)	+	+	+	+	+	-	+	-	+	+	+	+	+	-	-	+	-	+	+	-	-	-	+	-	+	+	-	-	+	+	-	-	-	+	-
Recurrent respiratory infections (HP:0002205)	+	+	+	-	+	+	+	+	+	-	na	+	-	-	+	+	+	+	+	+	+	+	+	+	+	+	+	-	+	-	-	-	+	+	-
Seizures (HP:0001250)	+	+	+	-	+	+	+	-	+	-	+	+	-	-	+	+	-	-	-	-	+	-	+	-	+	-	-	+	+	-	+	-	-	+	-
Treatment-refractary seizures (HP:0032867)	+	-	+	-	+	+	-	-	-	-	-	+	+	-	-	+	-	-	-	-	-	-	+	-	+	-	-	-	+	-	-	-	-	-	-
Gastroesophageal reflux (HP:0002020)	+	+	+	+	+	+	-	+	+	-	+	+	-	-	+	+	-	+	+	-	-	-	+	-	-	-	+	-	+	-	-	-	-	-	-
Constipation (HP:0002019)	+	+	+	+	+	-	+	-	+	+	+	-	+	+	+	+	-	+	+	+	+	-	-	+	-	+	+	+	-	+	-	-	+	+	-
Swallowing difficulties (HP:0002015)	+	+	+	+	+	+	+	+	+	+	+	+	+	+	+	+	-	-	+	-	-	-	+	-	+	-	+	-	+	+	-	-	-	-	-
Scoliosis (HP:0002650)	+	-	+	-	+	+	+	-	+	+	-	-	+	-	+	+	-	-	-	-	-	-	-	-	-	-	-	-	-	-	-	-	-	-	-
Cryptorchidism (HP:0000028)	-	-	+	-	-	-	-	na	-	-	-	+	+	-	-	+	-	-	-	-	-	-	-	-	+	+	-	-	-	-	-	-	-	-	-
Hypogenitalism (HP:0003241)	-	-	+	-	-	-	-	na	-	-	-	-	+	-	-	-	-	-	-	-	-	-	-	-	-	-	-	-	-	-	-	-	-	-	-
MECPDup score *	24	21	30	18	29	24	18	11	20	19	18	19	23	12	20	23	12	11	13	9	11	10	22	14	13	14	15	22	18	10	5	3	6	7	3

**Genetic variables**																																			
Maternally inherited duplication	+	-	+	+	+	+	+	+	+	+	+	+	+	-	-	+	-	+	+	+	+	+	+	-	+	+	+	+	+	-	-	+	-	-	+
Duplication size	0.6	5.8	0.6	13.5	2.9	2.8	0.9	0.5	0.5	0.5	14.3	1.3	0.7	2.7	0.6	0.2	0.4	0.4	0.4	0.2	0.4	0.4	0.3	0.8	0.4	0.8	0.4	0.4	0.4	0.3	0.3	0.3	8	0.6	8.0
Duplication location	na	Y	T	Y	Xp	Xp	T	T	T	T	Xp	T	T	18	T	T	na	T	T	T	T	T	T	T	T	T	T	T	T	T	T	T	T	T	T

F, female; M, male; na, not available; T, tandem duplication in the Xq28 region; X, X chromosome; Y, Y chromosome.

Supplemental Figure S1. Overall Study Framework and Data Pipeline.

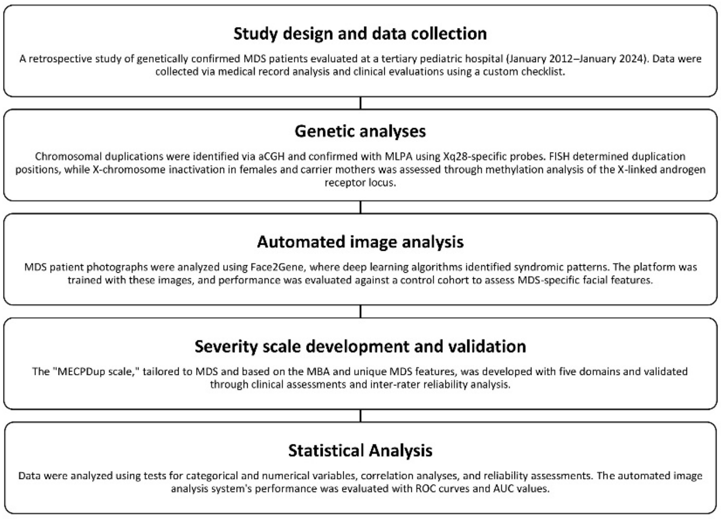

aCGH, array comparative genomic hybridization; AUC, area under the curve; FISH, fluorescence in situ hybridization; MLPA, multiplex ligation-dependent probe amplification; ROC, receiver operating curve.

Supplemental Figure S2. Scatter plot showing the correlation between the MECPDup score and age in males.

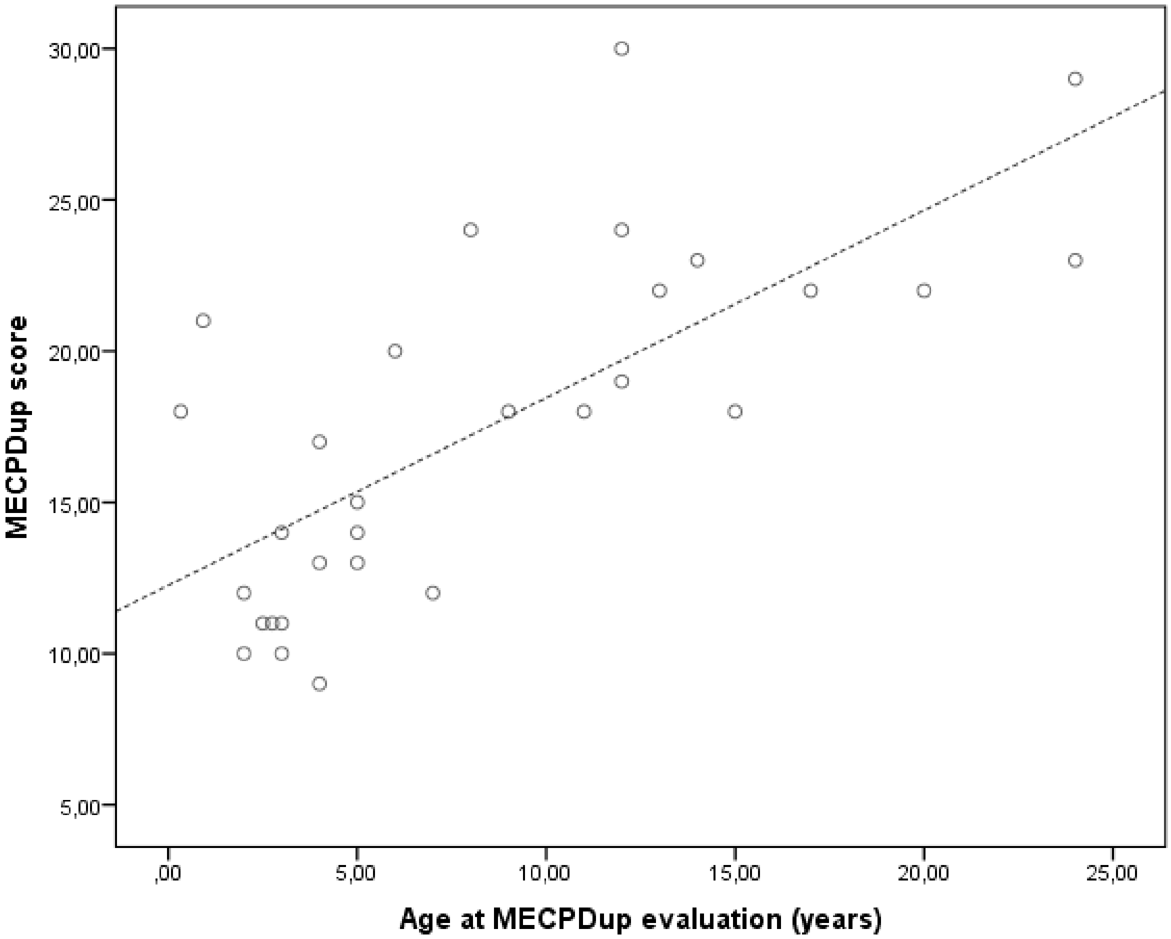

Supplement: Supplementary file 1 [file diagnostics-15-00010-s001.zip › diagnostics-3215922-supplementary.pdf]
